# Host Bloodmeal Identification in Cave-Dwelling *Ornithodoros turicata* Dugès (Ixodida: Argasidae), Texas, USA

**DOI:** 10.3389/fvets.2021.639400

**Published:** 2021-02-15

**Authors:** Rachel E. Busselman, Mark F. Olson, Viridiana Martinez, Edward Davila, Cierra Briggs, Devon S. Eldridge, Bailee Higgins, Brittany Bass, Thomas L. Cropper, Theresa M. Casey, Theresa Edwards, Pete D. Teel, Sarah A. Hamer, Gabriel L. Hamer

**Affiliations:** ^1^Department of Veterinary Integrative Biosciences, Texas A&M University, College Station, TX, United States; ^2^Department of Entomology, Texas A&M AgriLife Research, College Station, TX, United States; ^3^Department of Ecology and Conservation Biology, Texas A&M University, College Station, TX, United States; ^4^Department of Entomology, Cornell University, Ithaca, NY, United States; ^5^Department of Ecology and Evolutionary Biology, University of Tennessee, Knoxville, Knoxville, TN, United States; ^6^59th Medical Wing, Joint Base San Antonio, Lackland, San Antonio, TX, United States; ^7^Texas Parks and Wildlife Department, Government Canyon State Natural Area, San Antonio, TX, United States

**Keywords:** soft ticks, Argasidae, *Ornithodoros turicata*, blood meal, host identification

## Abstract

Tick-host bloodmeal associations are important factors when characterizing risks of associated pathogen transmission and applying appropriate management strategies. Despite their biological importance, comparatively little is known about soft tick (Argasidae) host associations in the United States compared to hard ticks (Ixodidae). In this study, we evaluated a PCR and direct Sanger sequencing method for identifying the bloodmeal hosts of soft ticks. We collected 381 cave-associated *Ornithodoros turicata* near San Antonio, Texas, USA, and also utilized eight colony-reared specimens fed artificially on known host blood sources over 1.5 years ago. We correctly identified the vertebrate host bloodmeals of two colony-reared ticks (chicken and pig) up to 1,105 days post-feeding, and identified bloodmeal hosts from 19 out of 168 field-collected soft ticks, including raccoon (78.9%), black vulture (10.5%), Texas black rattlesnake (5.3%), and human (5.3%). Our results confirm the retention of vertebrate blood DNA in soft ticks and advance the knowledge of argasid host associations in cave-dwelling *O. turicata*.

## Introduction

The identification of arthropod host-feeding patterns through bloodmeal analysis can provide key information for vertebrate host contact and pathogen transmission networks (1–4). Bloodmeal analysis methods based on the detection of vertebrate DNA left in the residual bloodmeal are widely used across diverse arthropod taxa. For example, reservoir hosts of *Leishmania* were identified by studying previous bloodmeals of sand flies (5), and host-feeding patterns in mosquitoes allowed for an enhanced understanding of the reservoirs of West Nile virus (6, 7). Molecular analysis of bloodmeals has also been used to identify a broad host community for *Culicoides*, vectors of avian Haemosporida infections (8), and of triatomines, vectors of *Trypanosoma cruzi*, agent of Chagas disease (9).

Bloodmeal analysis, applied to ticks, has repeatedly been associated with limited success (10, 11), likely owing to DNA degradation during the molt and many months since prior bloodmeal acquisition. Given their importance as vectors of human pathogens, several studies have conducted bloodmeal analysis of hard ticks (Ixodidae), identifying vertebrate hosts in 20–93% of analyzed ticks (12, 13). Given the challenges of PCR-Sanger sequencing-based bloodmeal analysis of hard ticks, alternative strategies have been evaluated to identify bloodmeal hosts, including analysis of the variation in stable isotopes in fed ticks (10, 14, 15), reverse line blot (16, 17), and proteomics (18). In comparison, relatively few studies have attempted to identify the bloodmeal hosts of argasid ticks (soft ticks).

*Ornithodoros turicata*, found in the southwestern United States and Florida (19), is a vector of human and animal pathogens. *O. turicata* is a known vector of tick-borne relapsing fever caused by *Borrelia turicatae* (20), and is also a putative vector for transmission of African swine fever virus, an emerging disease in Africa, Europe, and most recently Asia (21, 22). This DNA virus is transmitted by soft ticks of the *Ornithodoros* genus and is highly pathogenic to domestic swine (23). While African swine fever has yet to be detected in the U.S., recent studies have identified *O. turicata* as a most likely vector should the virus reach the US (24, 25).

*Ornithodoros turicata* is found in caves or burrows occupied or visited by diverse vertebrate hosts (19). Larvae, nymphs and adults attach, blood-feed, and drop from a host quickly (typically 15–20 min); thus, they are seldom collected from hosts during blood feeding events, complicating knowledge of tick-host associations (26). Further, they can survive for years between bloodmeals as nymphs and adults (27).

Identification of the host community that supports *O. turicata* populations could be useful in providing an ecological basis for vector control and disease management. We previously conducted a bloodmeal analysis study using quantitative PCR for the identification of vertebrates in experimentally fed *O. turicata* (28). The results demonstrated vertebrate DNA could be detected 330 days post-feeding and through multiple molts, suggesting longer retention of bloodmeal DNA in soft ticks compared to hard ticks. The current study builds on these prior results by conducting a PCR-Sanger sequencing bloodmeal analysis protocol on *O. turicata* fed experimentally on known hosts as well as field-collected specimens from cave environments in Texas near the location of recent outbreaks of TBRF in humans (29, 30).

## Materials and Methods

### Tick Collections

**Figure 1:**
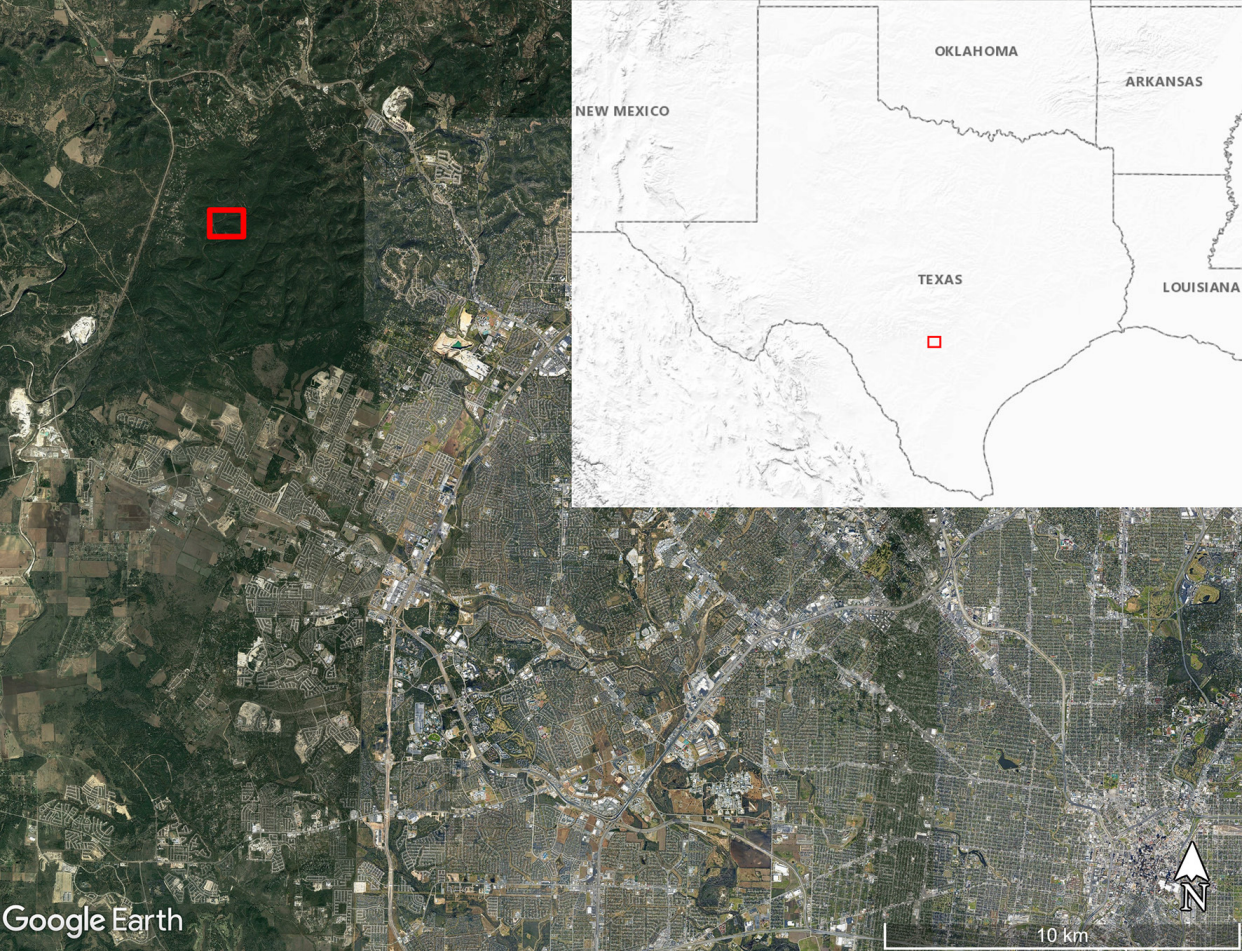
Map of soft tick collection location along Government Canyon Creek (red box) inside Government Canyon State Natural Area on the northwest side of San Antonio, Texas, USA. Map made using Google Earth Pro version 7.3.3.7786. Inset map shows the United States Geological Survey Shaded Relief map of the region around Texas with the map boundary near San Antonio outlined with the red box.

**Figure 2:**
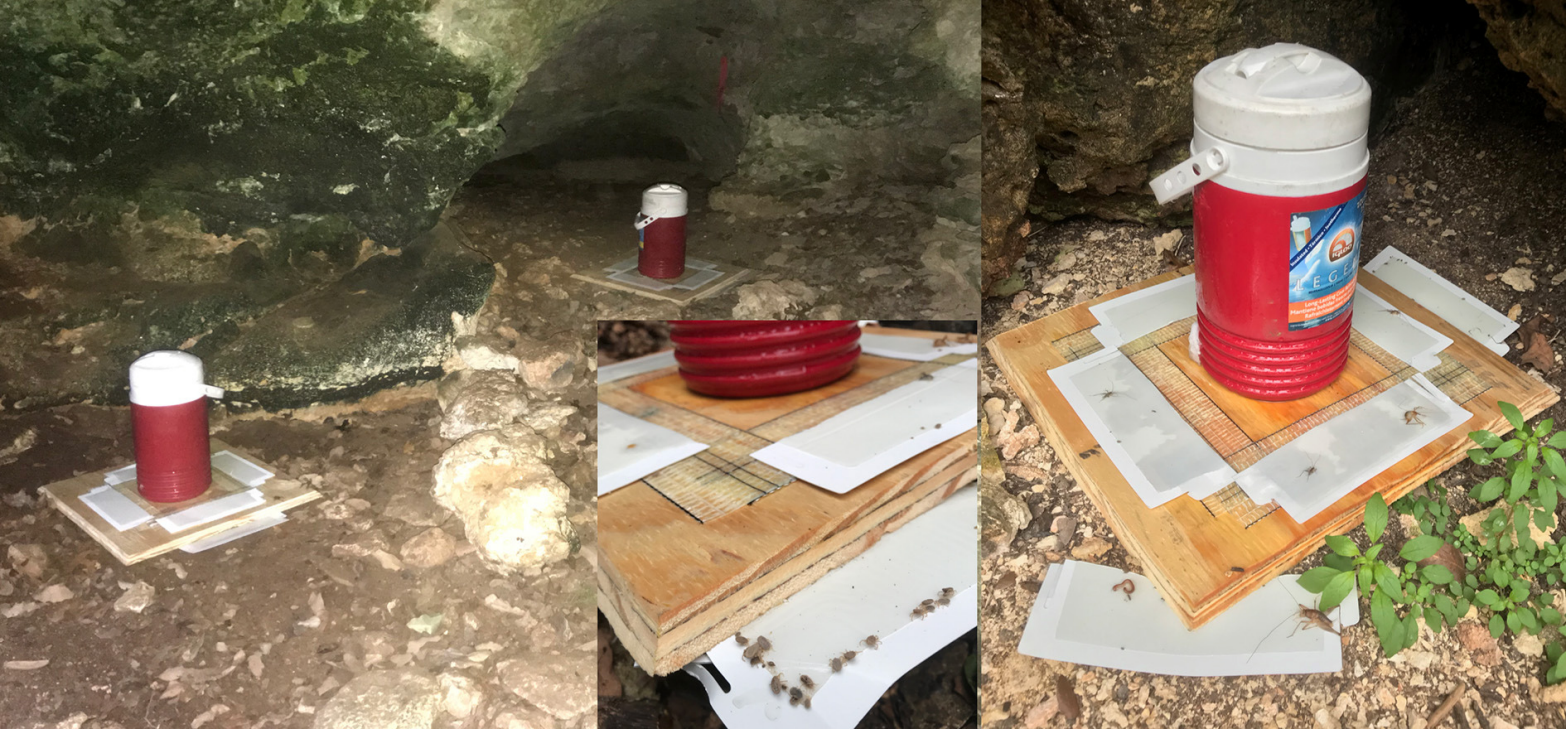
Pictures of dry ice-baited soft tick traps deployed inside and outside of caves in Government Canyon State Natural Area, Texas, USA. (**A**, left) Soft tick traps were placed with a cooler full of dry ice in the mouths of caves, and the cooler spout was left open for dry ice sublimation. (**B**, bottom middle) *O. turicata* soft ticks can be seen attached to the perimeter of the sticky tape. (**C**, right) Additional portions of sticky tape were added to parts of the perimeter of the trap to enhance collections.

We obtained eight adult *O. turicata* specimens (five male, three female) from an established colony at Texas A&M University, previously described (31). The feeding history of colony *O. turicata* was known only to one author (PDT) while the rest of the authors remained blinded to the bloodmeal species identification. We collected soft ticks from three caves in Government Canyon State Natural Area, San Antonio, TX, USA, (Lat: 29.549316, Lon:−98.764715) in March 2019 (Figure 1). Seven dry-ice baited sticky traps were set out inside or near the openings of three caves over 2 days for 18 and 21.5 h, respectively (Figure 2). These caves are closed to park visitors and were selected based on a previous study which demonstrated robust *O. turicata* populations in these caves (31). Traps consisted of 1.9L coolers filled with dry ice (cooler spout open) bolted through the bottom to a 0.41 m^2^ untreated 3-ply pine plywood with edges cut at 45° angles to improve tick access to the surface of the board. Double sided carpet tape (Roberts, Boca Raton, FL) was applied to the surface of the board, and insect glue boards (Bell Laboratories, Madison, WI) were cut into strips and applied to the carpet tape and under the corners of the plywood board. The ticks were removed from the sticky tape on-site and placed into ethanol-filled 1.5 ml tubes; each vial contained 3–10 ticks depending on the number of ticks caught at each trap each day. Samples were transported to the laboratory and stored at 4°C until DNA extraction.

### Tick Processing and DNA Extraction

*O. turicata* specimens were measured, identified to species and life stage by morphological features, and sexed if adults (32). A subset of 124 ticks were photographed to serve as a reference for confirming life stage and sex (Supplemental Data Sheet 1). Photos were taken early in the identification process and throughout tick cataloging as needed when life stage or sex was difficult to determine. We attempted to record the size of the bloodmeal based on the shape of the abdomen and presence of blood; however, the storage in ethanol produced a dark red color in all specimens and visual bloodmeal scoring was not reliable.

The first 20 ticks collected at each site, followed by equal numbers of the largest ticks from each cave (based on length), were processed further using the following methods until a threshold of 40% of the total number of collected ticks had been processed. To minimize exogenous DNA on the exterior surface of ticks, ticks were washed in ethanol for 5 s, then a 10% bleach solution for 15 s, and finally two consecutive 15 s DNA-free water rinses immediately after (10, 33, 34). On a sterile microscope slide over ice, the legs were then removed, placed in ethanol, and stored at −40°C to decrease the amount of tick DNA processed and to preserve samples of each tick for future use. The tick bodies were placed in clean tubes, flash-frozen in liquid nitrogen, and crushed with a sterile pestle (Wards Sciences, Rochester, NY), which was discarded after each use. The crushed tissue was then lysed, and DNA was extracted using the MagMAX CORE Nucleic Acid Purification Kit (Applied Biosystems ThermoFisher Scientific, Waltham, Massachusetts) according to the manufacturer's protocol. Negative and positive controls, including blood from sheep, tiger, and crane (vertebrate species not expected to occur around the cave environment), were included during DNA extraction procedures. The eluted tick DNA from each tick was stored in two tubes at −40°C until PCR amplification. For a small subset of samples from two early extractions (*n* = 6), we quantified DNA using a spectrophotometer (Epoch, BioTek Instruments, Inc.) to confirm the presence of DNA in the extracted samples (average = 32.39 ng/μL, range = 9.07–68.07 ng/μL).

### PCR Amplification and Sanger Sequencing

DNA extracts from ticks were subjected to multiple vertebrate barcoding primers targeting different genes in an iterative process to identify the most successful primers (Supplementary Table 1). The Failsafe PCR Enzyme Mix with PreMix E (Epicenter Biotechnologies, Madison, WI) and primer pairs from Integrated DNA Technologies (IDT) were used for PCR amplification. Primers were used at varying concentrations of 0.33 or 0.4 μM (see Supplementary Table 1) in 25 μL reactions, including 2 μL of tick DNA. PCR products were visualized on e-gels (ThermoFisher Scientific, Waltham, Massachusetts) and all products were purified by Exo-SAP-IT (Applied Biosystems, Foster City, CA) and sequenced in forward directions by Eton Biosciences (San Diego, California). Sequences were trimmed to at least 215 base pairs long, and chromatographs were manually scrutinized for quality. Sequences were blasted to the NCBI database using Geneious software (Newark, New Jersey) to identify the closest match of the unknown sequence to a known organism. Sequences with >90% similarity were interpreted as a match, in which case the bloodmeal host was identified. All samples that produced sequences with double peaks were re-run with the “mammal c” primer pair. Because of the chance for contamination of samples with human DNA, any sample that produced a sequence that matched to human was tested a second time with an independent PCR, and in these cases, two matches to the same species were needed to confirm a result.

## Results

### Colony Ticks

Bloodmeal analysis was conducted on eight *O. turicata* from a colonized population with known prior bloodmeals, with personnel conducting the molecular work blinded to the vertebrate species. We successfully detected chicken (*Gallus gallus*) in an adult tick 1,105 days (last fed 27 January 2016) post bloodmeal and pig (*Sus scrofa*) in an adult tick 622 days (last fed 24 May 2017) days post bloodmeal. We were unable to obtain a PCR amplicon or sequence from six ticks that had fed 622–1,109 days post-bloodmeal (last fed on dates through January 23, 2016 to May 24, 2017).

### Field-Collected Ticks

A total of 381 soft ticks were collected in March 2019, and all were identified by morphology as *O. turicata*. Mad Crow Cave yielded the highest number of soft ticks trapped (*n* = 184), followed by Bone Pile Cave (*n* = 109) and Little Crevice Cave (*n* = 88). We identified 32 females, 55 males, 285 nymphs, four adults that were damaged and unable to be sexed, and five other specimens that were damaged and we were unable to determine either sex or life stage (Table 1). The average length of males was 3.53 mm (*n* = 54, *SD*: 0.98), 5.28 mm for females (*n* = 32, *SD*: 1.70), and 2.23 mm (*n* = 284; *SD*: 0.82) for nymphs. One male and one nymph were damaged and unable to be measured.

**Table 1:** Demographic data of field-collected *Ornithodoros turicata* collected from Government Canyon State Natural Area, San Antonio, TX, 2019.

**Cave name**	**Adult male**	**Adult female**	**Adult unknown**	**Life stage**	**Nymphs**	**Total**
				**unknown**		
Mad Crow Cave	20 (10.9%)	13 (7.1%)	1 (0.5%)	5 (2.7%)	145 (78.8%)	184
Bone Pile Cave	10 (9.2%)	14 (12.8%)	1 (0.9%)	0	84 (77.1%)	109
Little Crevice Cave	25 (28.4%)	5 (5.7%)	2 (2.3%)	0	56 (63.6%)	88

A subset of 168 field-collected ticks were processed for bloodmeal analysis. Most PCR primers amplified exclusively *Ornithodoros* sp. DNA, including the primer pairs mammal c, 0066/0067, 0035/0049, 0033/0049, and Herp/BM1. The primer pair which had the best success at minimizing tick DNA amplification and maximizing vertebrate DNA amplification was “mammal c” targeting a 395 base pair region of cytochrome b (4, 35). The bloodmeals from 19 ticks (11.3%) were identified to species using the “mammal c” primer pair (Table 2). Of this subset, 15 ticks (78.9%) contained raccoon (*Procyon lotor*) DNA, two ticks (10.5%) contained black vulture (*Coragyps atratus*) DNA, one tick (5.3%) contained black-tailed rattlesnake (*Crotalus molossus*) DNA, and one tick (5.3%) contained human (*Homo sapiens*) DNA. Of the 168 field-collected samples subjected to bloodmeal analysis, 69 were adults (39 male, mean size = 3.62 mm; 26 female, mean size = 5.67 mm), 97 were nymphs (mean size = 2.64 mm, *n* = 96 as one tick length was unreliable), and two were unable to be determined. The 19 ticks with identifiable bloodmeals included four males (mean size = 3.48 mm), one female (5.6 mm), and 14 nymphs (mean size = 2.96 mm).

**Table 2:** Demographic data of ticks and their identified bloodmeal sources.

**Cave/location name**	**Tick ID number**	**Life stage**	**Sex**	**Length (mm)**	**Vertebrate bloodmeal result (% match)**
Colony	A2	A	M	3.7	*Gallus gallus* (99.7%)
Colony	B2	A	F	7	*Sus scrofa* (95.4%)
Mad Crow Cave	ST-02A	N	U	2	*Procyon lotor* (92%)
Mad Crow Cave	ST-02B	N	U	3.5	*Procyon lotor* (100%)
Mad Crow Cave	ST-05B	N	U	4.3	*Coragyps atratus* (99.3%)
Bone Pile Cave	ST-18E	N	U	2.5	*Procyon lotor* (90%)
Bone Pile Cave	ST-19B	N	U	3	*Procyon lotor* (96%)
Bone Pile Cave	ST-19C	N	U	2	*Procyon lotor* (99.7%)
Bone Pile Cave	ST-20B	N	U	2.5	*Procyon lotor* (100%)
Bone Pile Cave	ST-21E	A	M	3	*Procyon lotor* (100%)
Bone Pile Cave	ST-22B	A	M	4.5	*Crotalus molossus* (98.9%)
Bone Pile Cave	ST-40B	N	U	2.5	*Procyon lotor* (96.2%)
Bone Pile Cave	ST-46B	N	U	2.1	*Procyon lotor* (90.3%)
Little Crevice Cave	ST-65E	N	U	3	*Homo sapien* (99.7%)
Little Crevice Cave	ST-68A	A	M	2	*Procyon lotor* (100%)
Mad Crow Cave	ST-72H	N	U	3.25	*Coragyps atratus* (100%)
Mad Crow Cave	ST-74E	A	F	5.6	*Procyon lotor* (99.7%)
Mad Crow Cave	ST-78C	N	U	4.8	*Procyon lotor* (99.7%)
Mad Crow Cave	ST-78I	A	M	4.4	*Procyon lotor* (99.7%)
Mad Crow Cave	ST-80B	N	U	3	*Procyon lotor* (99.7%)
Mad Crow Cave	ST-80H	N	U	3	*Procyon lotor* (95.1%)

*All bloodmeals were identified using the mammal c primer pair. A, Adult; N, Nymph; M, Male; F, Female; U, Unknown*.

## Discussion

This study builds on prior results, which demonstrated that vertebrate DNA detected by quantitative PCR in prior bloodmeals of *O. turicata* persists for long periods post-feeding and through molts (27, 31). In the current study, we adopted PCR and direct Sanger sequencing and confirmed that, for experimentally fed ticks in the laboratory, we were able to detect bloodmeals that were up to 1,105 days old. However, a challenge encountered by the molecular approach used in this study was that *Ornithodoros* sp. DNA was amplified consistently using five different primer pairs (mammal c, 0066/0067, 0035/0049, 0033/0049, and Herp/BM1). Amplification of vector DNA has not been an issue during mosquito bloodmeal analysis studies, which served as a main source of bloodmeal primers used in this study (3, 34, 36). Many of the chromatographs from the sequences suggested double-nucleotide peaks in the amplicons that matched to *Ornithodoros* sp., and repeated PCRs with the same or different primers were unable to resolve the amplified sequences. We suspect this non-target amplification of tick DNA is attributed to the barcoding primer design that minimizes non-target amplification of Insecta but perhaps not Ixodida. The size of the argasid tick genome (1.2 Gbp) is also 2.2x larger than the genome of *Culex pipiens* (0.54 Gbp), a common mosquito in which bloodmeal primers are developed, which further increases the opportunity for non-target amplification (35, 37, 38).

Despite the challenges posed by the non-target amplification of soft tick DNA, we were still able to produce repeatable bloodmeal host identification results in 19 samples. We suspect the success in the vertebrate ID in these samples was possible when sufficient blood was present. The most common vertebrate ID for these cave-dwelling soft ticks was raccoon, followed by black vulture, black-tailed rattlesnake, and human. The presence of these species in these caves was confirmed during a prior camera trapping study (31). Although these caves are off-limits to the public, camera traps documented unauthorized human access to these caves, supporting the potential for a human bloodmeal in this study and also identifying a risk associated with exposure to *B. turicatae*, which has been documented for central Texas (29, 39, 40). A study in Iowa documented large infestations of the soft tick, *Carios kelleyi*, in human dwellings using capillary electrophoresis to conduct bloodmeal analysis and identified one nymphal soft tick that had fed on a female human (1). Additionally, *Argas cooleyi* invaded a hospital in Arizona from their resident bird nests outside and fed on humans, with 17% of the analyzed bloodmeals belonging to humans (41). These studies, along with our study of a wild *O. turicata* population, indicate the importance of studying soft ticks and their potential associations with humans as hosts.

This cross-sectional study also informs the population structure of *O. turicata* in the caves of the region. Of the 381 collected soft ticks, 74.8% were found to be in the nymphal stage and no larvae were collected. These skewed demographics of the soft tick community composition illustrate a large proportion of immature ticks in early March compared to adults. Some soft tick species molt to future nymphal instars based on ambient temperature, and in some species, larvae do not need to feed on a bloodmeal, and receive all the nutrients they need to molt into first instar nymphs from the egg (26). However, in colony, *O. turicata* have been recorded to feed between larval and nymphal stages, and nymphs reared in similar environmental conditions can molt to adults as either 4, 5, or 6th nymphal instars (42). Due to the limited literature available on soft tick ecology, this study may indicate that in spring months, nymphs are more abundant than adults. Alternately, larvae may have been present in the caves yet less likely to be trapped using the dry ice/sticky trap stations that we deployed. Population demographic data is key in understanding the natural cycles of soft tick populations.

Limited prior studies have documented vertebrate host feeding patterns of soft ticks collected in the field. A study in Portugal performed bloodmeal analysis on *Ornithodoros erraticus* collected in two pig pens and were able to identify vertebrate hosts including pigs, humans, bovines, sheep, rodents, and birds through bloodmeal analysis in 23% of the analyzed ticks (4). We used many of the same primer pairs from this previous study, and the lower success of the bloodmeal identification was likely also due to non-target amplification of tick DNA.

The challenges of non-target amplification of tick DNA could be resolved by multiple modifications in the future. All specimens collected in this study were host-seeking as they approached the CO_2_-baited traps. Accordingly, very few likely had fresh bloodmeals, which made it difficult to distinguish bloodmeal contents in the abdomen, especially when ethanol added a red coloration to all specimens. Future studies processing field-collected soft ticks should consider the use of morphological features such as the size and depth of inter-mammillary grooves to judge the state of repletion. A future sampling approach could use an aspirator (43) which would increase the chances of obtaining specimens with fresh bloodmeals. One modification would be to specifically design primers to avoid tick genomes and amplify exclusively vertebrate DNA (35, 44). Another modification could be to insert a clone of the amplicon into a bacterial vector, and then select several colonies per sample to sequence, in hopes of detecting the vertebrate host sequence even if tick DNA was preferentially amplified. This technique is used routinely in bloodmeal analysis studies (45), although this method is labor intensive and limited in resolution. A third option would be to perform amplicon deep sequencing, which would provide thousands of sequences of each amplicon and is an approach recently adopted for arthropod bloodmeal analysis studies (46–48). This method of metabarcoding and deep sequencing would be advantageous given soft tick biology, including multiple bloodmeals obtained during immature development and multiple gonotropic cycles of adults. The amplicon deep sequencing approach would allow the ID of not just the most recent bloodmeal but also the potential to detect prior vertebrate bloodmeals (49, 50).

Given the continued emergence of human and animal diseases vectored by soft ticks, further studies of the ecology of argasid ticks- including their vertebrate host associations- are critical for informing tick-host-pathogen transmission networks, vector management efforts, and disease risk assessment.

## Data Availability Statement

The datasets presented in this study can be found in online repositories. The names of the repository/repositories and accession number(s) can be found at: https://hdl.handle.net/1969.1/191880, OAKTrust, Texas A&M University Libraries.

## Author Contributions

PT, SH, and GH conceived study, supervised field, and lab work. RB, VM, ED, TE, TLC, TMC, and GH conducted field collections. RB, MO, VM, ED, CB, DE, BH, and BB conducted lab work. PT provided colony ticks. RB, VM, and ED wrote initial manuscript. All authors contributed to revisions.

## Conflict of Interest

The authors declare that the research was conducted in the absence of any commercial or financial relationships that could be construed as a potential conflict of interest.
