# Supplementary material for: Host Bloodmeal Identification in Cave-Dwelling Ornithodoros turicata Dugès (Ixodida: Argasidae), Texas, USA
Source: Front Vet Sci. 2021 Feb 15;8:639400. doi: 10.3389/fvets.2021.639400 (PMC7917080; doi:10.3389/fvets.2021.639400)

Supplementary Figure 1: Images of soft ticks used in the blood meal analysis. **(A&B)** Adult female soft tick from colony, ventral and dorsal view respectively (ST-B2). **(C)** Adult male soft tick caught in traps in Bone Pile Cave (ST-22B). **(D)** Nymph caught in soft tick traps in Mad Crow Cave (ST-02A).
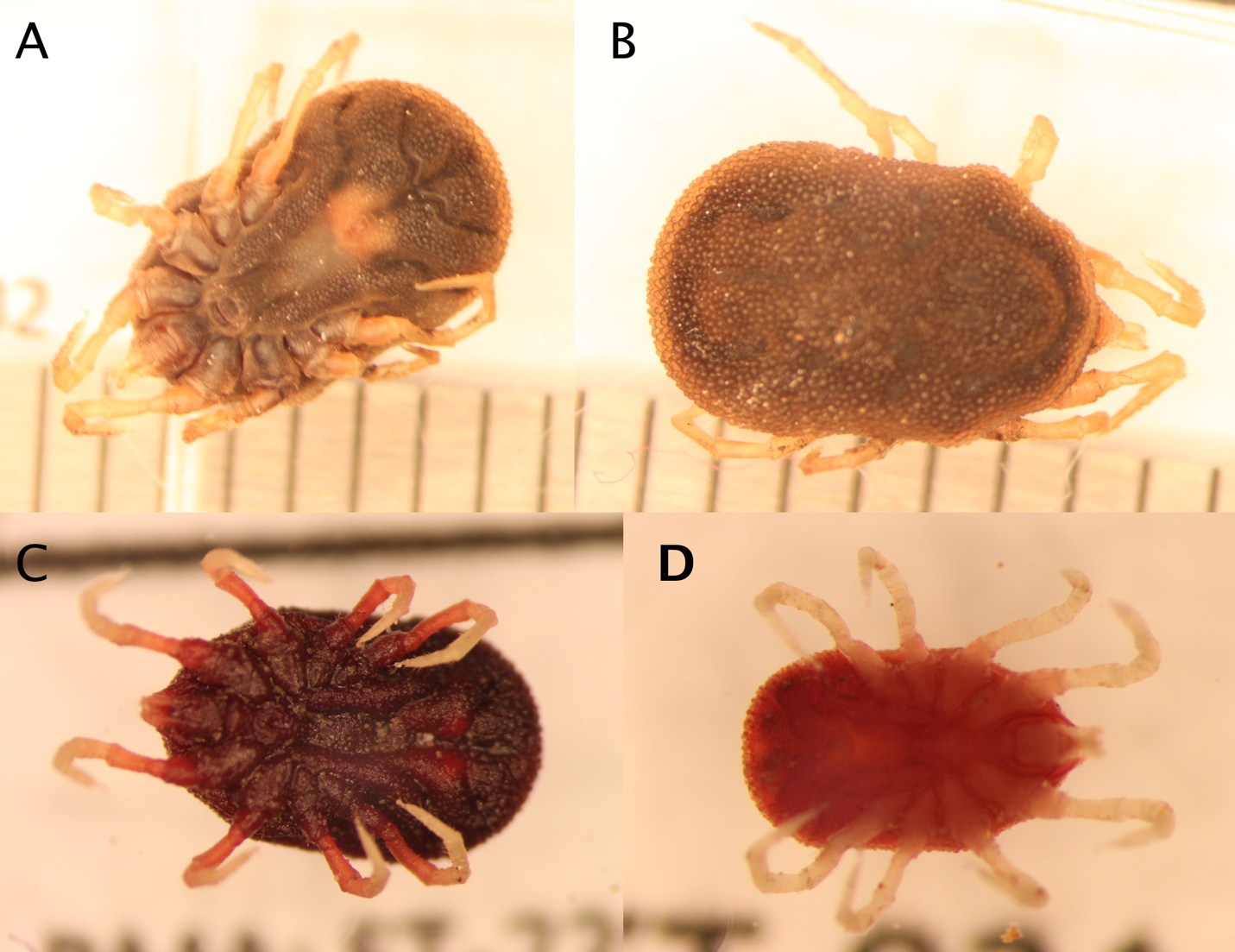

Supplement: Supplementary file 1 [file Data_Sheet_1.DOCX]
