# Supplementary material for: Host Bloodmeal Identification in Cave-Dwelling Ornithodoros turicata Dugès (Ixodida: Argasidae), Texas, USA
Source: Front Vet Sci. 2021 Feb 15;8:639400. doi: 10.3389/fvets.2021.639400 (PMC7917080; doi:10.3389/fvets.2021.639400)
Supplement: Supplementary file 2 [file Table_1.DOCX]

Supplementary Table 1. Published vertebrate barcoding primers used in the current study to identify the vertebrate species previously fed on by blood-engorged *Ornithodoros turicata*.

| Gene Target | Primer Name | Primer 5’ -> 3’ | PCR Cycling | Amplicon Size (bp) | Final Primer Concentration | References |
| --- | --- | --- | --- | --- | --- | --- |
| COI (cytochrome c oxidase) | VF1_t1 (1) | 5’-TGTAAAACGACGGCCAGTTCTCAACCAACCACAAAGACATTGG-3’ | 94°C for 1 min, (5 cycles of 94°C for 30s , 50°C for 40 seconds, and 72°C for 1 min, (35 cycles of 94°C for 30s, 54°C for 40s, and 72°C for 1 min,) and final 72°C for 10 min | 648 | 0.33 µM * | (Ivanova et al., 2006; Ivanova et al., 2007; Kent, 2009; Gariepy et al., 2012) |
|  | VF1d_t1 (1) | 5’-TGTAAAACGACGGCCAGTTCTCAACCAACCACAARGAYATYGG-3’ |  |  |  |  |
|  | VF1i_t1 (2) | 5’-TGTAAAACGACGGCCAGTTCTCAACCAACCAIAAIGAIATIGG-3’ |  |  |  |  |
|  | VR1d_t1 (1) | 5’-CAGGAAACAGCTATGACTAGACTTCTGGGTGGCCRAARAAYCA-3’ |  |  | 0.33 µM * |  |
|  | VR1_t1 (1) | 5’-CAGGAAACAGCTATGACTAGACTTCTGGGTGGCCAAAGAATCA-3’ |  |  |  |  |
|  | VR1i_t1 (2) | 5’-CAGGAAACAGCTATGACTAGACTTCTGGGTGICCIAAIAAICA-3’ |  |  |  |  |
| cytochrome b | BM_1 | 5’-CCCCTCAGAATGATATTTGTCCTCA-3’ | 3.5 minutes at 95°C, (36 cycles 30 seconds at 95°C, 50 seconds at 60°C, 40 seconds at 72°C), and final 5 minutes at 72°C | 358 | 0.33 µM | (Hamer et al., 2009) |
|  | BM_2 | 5’-CCATCCAACATCTCAGCATGATGAAA-3’ |  |  | 0.33 µM |  |
| cytochrome b | Herp_f | 5’-GCHGAYACHWVHHYHGCHTTYTCHTC-3’ | 2 minutes 95°C, 55 cycles 45 seconds at 94°C, 50 seconds at 50°C, 1 minute at 72°C, and final 7 minutes at 72°C | 228 | 0.33 µM | (Cupp et al., 2004; Hamer et al., 2009) |
|  | Herp_r | 5’-CCCCTCAGAATGATATTTGTCCTCA-3’ |  |  | 0.33 µM |  |
| vertebrate 18S rDNA | 0033 | 5’-TTCTAGAGCTAATACATGCCGA-3’ | 94°C for 4 min, (50 cycles of 30 s at 94°C, 30 s at 59°C, and 30 s at 72°C,) and finish 5 min at 72°C | 150 (mammals) 120 (birds) | 0.33 µM | (Pichon et al., 2003) |
| vertebrate 18S rDNA | 0049 | 5’- YCGAGGTTATCTAGAGTCACC-3’ | 94°C for 4 min, (50 cycles of 30 s at 94°C, 30 s at 59°C, and 30 s at 72°C,) and finish 5 min at 72°C | 150 (mammals) 120 (birds) | 0.33 µM | (Pichon et al., 2003; Allan et al., 2010) |
| vertebrate 18S rRNA | 0035 | 5’-TTCTAGAGCTAATACATGCCRA-3’ |  |  | 0.33 µM | (Allan et al., 2010) |
| vertebrate 18S rDNA | 0066 | 5’-ACCTGGTTGATCCTGCCA-3’ | 94°C for 4 min, (35 cycles of 30 seconds at 94°C, 30 seconds at 57°C, and 45 seconds at 72°C,) and final 5 min at 72°C | 400 (mammals) 350 (birds) | 0.33 µM | (Pichon et al., 2003; Allan et al., 2010) |
| vertebrate 18S rDNA | 0067 | 5’-TACCATCGAAAGTTGATAGGG-3’ | 94°C for 4 min, (35 cycles of 30 seconds at 94°C, 30 seconds at 57°C, and 45 seconds at 72°C,) and final 5 min at 72°C | 400 (mammals) 350 (birds) | 0.33 µM | (Pichon et al., 2003; Allan et al., 2010) |
| cytochrome b | avian a_f | 5’-GAC TGT GAC AAA ATC CCN TTC CA-3’ | 3.5 minutes at 95°C, (36 cycles 30 seconds at 95°C, 50 seconds at 60°C, 40 seconds at 72°C), and final 5 minutes at 72°C | 508 | 0.33 µM | (Cicero and Johnson, 2001; Molaei et al., 2006; Hamer et al., 2009; Palma et al., 2013) |
|  | avian a_r | 5’-GGT CTT CAT CTY HGG YTT ACA AGA C-3’ |  |  | 0.33 µM |  |
| cytochrome b | avian b_f | 5’-CCC TCA GAA TGA TAT TTG TCC TCA-3’ | 3.5 minutes at 95°C, (36 cycles 30 seconds at 95°C, 50 seconds at 60°C, 40 seconds at 72°C), and final 5 minutes at 72°C | 515 | 0.33 µM | (Sorenson et al., 1999; Molaei et al., 2006; Palma et al., 2013) |
|  | avian b_r | 5’-CCT CAG AAK GAT ATY TGN CCT CAK GG-3’ |  |  | 0.33 µM |  |
| cytochrome b | mammal a_f | 5’-CGA AGC TTG ATA TGA AAA ACC ATC GTT G-3’ | 3.5 minutes at 95°C, (36 cycles 30 seconds at 95°C, 50 seconds at 60°C, 40 seconds at 72°C), and final 5 minutes at 72°C | 772 | 0.33 µM | (Molaei et al., 2006; Hamer et al., 2009; Palma et al., 2013) |
|  | mammal a_r | 5’-TGT AGT TRT CWG GGT CHC CTA-3’ |  |  | 0.33 µM |  |
| cytochrome b | mammal b_f | 5’-GCG TAC GCA ATC TTA CGA TCA A-3’ | 3.5 minutes at 95°C, (36 cycles 30 seconds at 95°C, 50 seconds at 60°C, 40 seconds at 72°C), and final 5 minutes at 72°C | 195 | 0.33 µM | (Molaei et al., 2006; Palma et al., 2013) |
|  | mammal b_r | 5’-CTG GCC TCC AAT TCA TGT GAG-3’ |  |  | 0.33 µM |  |
| cytochrome b | mammal c_f | 5’-CCA TCC AAC ATC TCA GCA TGA TGA AA-3’ | 3.5 minutes at 95°C, (36 cycles 30 seconds at 95°C, 50 seconds at 60°C, 40 seconds at 72°C), and final 5 minutes at 72°C | 395 | 0.4 µM | (Molaei et al., 2006; Palma et al., 2013) |
|  | mammal c_r | 5’-GCC CCT CAG AAT GAT ATT TGT CCT CA-3’ |  |  | 0.4 µM |  |
| COI (cytochrome c oxidase subunit I) | Mod_RepCOI_F | 5’-TNTTYTCMACYAACCACAAAGA -3’ | 94 ̊C for 3 min, 40 cycles of 94 ̊C for 40s, 48.5 ̊C for 30s, and 72 ̊C for 60s, and final 7 minutes at 72 ̊C | 664 | 0.33 µM | (Reeves et al., 2018) |
|  | Mod_RepCOI_R | 5’-TTC DGG RTG NCC RAA RAA TCA-3’ |  |  | 0.33 µM |  |
| COI (cytochrome c oxidase subunit I) | 7194 | 5’-CGM ATR AAY AAY ATR AGC TTC TGA Y-3’ | 94 ̊C for 3 min, 40 cycles of 94 ̊C for 40s, 48.5 ̊C for 30s, and 72 ̊C for 60s, and final 7 minutes at 72 ̊C | 395 | 0.33 µM | (Reeves et al., 2018) |

* This calculation is the final primer concentration of all three forward primers (VF1_t1, TF1d_t1, VF1i_t1) and all three reverse primers (VR1d_t1, VR1_t1, VR1i_t1) mixed together in a 1:1:2 ratio, respectively.

**References:**

Allan, B.F., Goessling, L.S., Storch, G.A., and Thach, R.E. (2010). Blood meal analysis to identify reservoir hosts for *Amblyomma americanum* ticks. *Emerg Infect Dis* 16(3)**,** 433-440. doi: 10.3201/eid1603.090911.

Cicero, C., and Johnson, N.K. (2001). Higher-level phylogeny of new world vireos (aves: vireonidae) based on sequences of multiple mitochondrial DNA genes. *Mol Phylogenet Evol* 20(1)**,** 27-40. doi: 10.1006/mpev.2001.0944.

Cupp, E.W., Zhang, D., Yue, X., Cupp, M.S., Guyer, C., Sprenger, T.R., et al. (2004). Identification of reptilian and amphibian blood meals from mosquitoes in an eastern equine encephalomyelitis virus focus in Central Alabama. *Am J Trop Med Hyg* 7(1)**,** 272-276. doi: 10.1056/NEJMp1914328.

Gariepy, T.D., Lindsay, R., Ogden, N., and Gregory, T.R. (2012). Identifying the last supper: utility of the DNA barcode library for bloodmeal identification in ticks. *Mol Ecol Resour* 12(4)**,** 646-652. doi: 10.1111/j.1755-0998.2012.03140.x.

Hamer, G.L., Kitron, U., Goldberg, T.L., Brawn, J.D., Loss, S.R., Ruiz, M.O., et al. (2009). Host Selection by *Culex pipiens* mosquitoes and West Nile virus amplification. *Am J Trop Med Hyg* 80(2)**,** 268-278. doi: 10.4269/ajtmh.2009.80.268.

Ivanova, N.V., Dewaard, J.R., and Hebert, P.D.N. (2006). An inexpensive, automation-friendly protocol for recovering high-quality DNA. *Mol Ecol Notes* 6(4)**,** 998-1002. doi: 10.1111/j.1471-8286.2006.01428.x.

Ivanova, N.V., Zemlak, T.S., Hanner, R.H., and Hebert, P.D.N. (2007). Universal primer cocktails for fish DNA barcoding. *Mol Ecol Notes* 7(4)**,** 544-548. doi: 10.1111/j.1471-8286.2007.01748.x.

Kent, R.J. (2009). Molecular methods for arthropod bloodmeal identification and applications to ecological and vector-borne disease studies. *Mol Ecol Resour* 9(1)**,** 4-18. doi: 10.1111/j.1755-0998.2008.02469.x.

Molaei, G., Andreadis, T.G., Armstrong, P.M., Anderson, J.F., and Vossbrinck, C.R. (2006). Host feeding patterns of *Culex* mosquitoes and West Nile virus transmission, Northeastern United States. *Emerg Infect Dis* 12(3)**,** 468-474. doi: 10.3201/eid1203.051004.

Palma, M., Lopes de Carvalho, I., Osorio, H., Ze-Ze, L., Cutler, S.J., and Nuncio, M.S. (2013). Portuguese hosts for *Ornithodoros erraticus* ticks. *Vector Borne Zoonotic Dis* 13(10)**,** 775-777. doi: 10.1089/vbz.2012.1070.

Pichon, B., Egan, D., Rogers, M., and Gray, J. (2003). Detection and identification of pathogens and host DNA in unfed host-seeking *Ixodes ricinus* L. (Acari: Ixodidae). *J Med Entomol* 40(5)**,** 723-731. doi: 10.1603/0022-2585-40.5.723.

Reeves, L.E., Gillett-Kaufman, J.L., Kawahara, A.Y., and Kaufman, P.E. (2018). Barcoding blood meals: New vertebrate-specific primer sets for assigning taxonomic identities to host DNA from mosquito blood meals. *PLOS Negl Trop Dis* 12(8)**,** e0006767. doi: 10.1371/journal.pntd.0006767.

Sorenson, M.D., Ast, J.C., Dimcheff, D.E., Yuri, T., and Mindell, D.P. (1999). Primers for a PCR-based approach to mitochondrial genome sequencing in birds and other vertebrates. *Mol Phylogenet. Evol* 12(2)**,** 105-114.
